# Patient-to-worker violence in Public hospitals in Tunisia

**DOI:** 10.1192/j.eurpsy.2023.1167

**Published:** 2023-07-19

**Authors:** A. Omrane, C. Harrathi, I. Mlouki, M. Ouerchefani, T. Khalfallah, S. EL Mhamedi, L. Bouzgarrou

**Affiliations:** 1 Occupational Medicine and Ergonomics; 2 Preventive and community medicine, Faculty of Medicine of Monastir Tunisia, Monastir, Tunisia

## Abstract

**Introduction:**

Compared with workers in other sectors, hospital workers are victims of high rates of non-fatal workplace assault injuries worldwide. Unfortunately, a large amount of these injuries is a result from violent acts committed by patients. International research has focused on perceived reasons for patient violence among physicians and patients.

**Objectives:**

To determine the prevalence and factors of patient-to worker violence in two hospitals in the central-eastern region of Tunisia.

**Methods:**

A cross-sectional bi-centric study was conducted in two teaching hospitals. This study included all healthcare workers of these hospitals. Data collection was performed by a structured self-administered questionnaire related to demographic and professional characteristics of participants. Participants were asked about violence where the perpetrators were patients using a questionnaire developed and validated by a Jordanian team to evaluate Violence. Beck’s Depression Inventory II and Rosenberg self-esteem scale were chosen to explore self-esteem issues and mood disorders. The Fagerstrom test for nicotine dependence was used to assess cigarette dependence. The Internet addiction test was used to evaluate internet addiction.

**Results:**

The response rate was of 19%. The mean age of the sample was 34.5 ±9.6 years. In work sit, 46.9% (n=239) reported being exposed to at least one incident of patient-to-worker violence. Exposure to verbal violence was reported by 92.1% of workers. Among the sample, 18.9% of participants reported feelings of low self-esteem. Asked about depressive symptoms, appetite problems were reported in 77.1% of cases. A significant association was found between patient-to-worker violence, nationality (p= 0.01) and occupation (p= 0.01) of respondents. Results showed also that patient-to-worker violence was significantly associated with smoking (p=0.043), the degree of cigarette addiction (≤10-3) and alcohol consumption (p=0.008). Mood disorders were associated to exposure to patient-to-worker violence. An increased risk to be exposure to incidents of physical violence was found among workers with depressive symptoms. Also, reporting physical violence was associated with self-esteem issues and feelings of worthlessness.

**Conclusions:**

Violence has become an alarming hazard in hospitals. This study showed the association between different factors and the exposure to patient-to-worker violence incidents in two hospitals. Being exposed to patient-to-worker violence has negative impacts. Some of type II violence’s consequences are to impact quality of life of workers, depression, psychological squeals, effectiveness of work and the decrease of quality of care. In order to reduce patient-to-worker violence, urgent prevention programs should be incorporated in hospitals.

**Disclosure of Interest:**

None Declared

